# Single-capillary endothelial dysfunction resolved by optoacoustic mesoscopy

**DOI:** 10.1038/s41377-025-02103-6

**Published:** 2026-01-03

**Authors:** Hailong He, Angelos Karlas, Nikolina-Alexia Fasoula, Chiara Fischer, Ulf Darsow, Michael Kallmayer, Juan Aguirre, Hans-Henning Eckstein, Vasilis Ntziachristos

**Affiliations:** 1 Chair of Biological Imaging, Central Institute for Translational Cancer Research (TranslaTUM), School of Medicine and Health & School of Computation, Information and Technology, Technical University of Munich, Munich, Germany; 2 Institute of Biological and Medical Imaging, Bioengineering Center, Helmholtz Zentrum München, Neuherberg, Germany; 3 DZHK (German Centre for Cardiovascular Research), partner site Munich Heart Alliance, Munich, Germany; 4 Clinic and Polyclinic for Vascular and Endovascular Surgery, TUM University Hospital, Hospital Rechts der Isar, Technical University of Munich, Munich, Germany; 5 Chair for Computer Aided Medical Procedures & Augmented Reality, Technical University of Munich, Munich, Germany; 6 Clinic for Vascular Surgery, Helios Klinikum München West, Munich, Germany; 7 Department of Dermatology and Allergy, Technical University of Munich, Munich, Germany; 8 Departamento de Tecnología Electrónica y de las Comunicaciones, Universidad Autónoma de Madrid, Madrid, Spain; 9 Instituto de Investigación Sanitaria de la Fundación Jiménez Díaz, Madrid, Spain

**Keywords:** Optical techniques, Applied optics

## Abstract

Microvascular endothelial dysfunction (MiVED) is an early marker of endothelial impairment, often preceding dysfunction in large arteries. Although MiVED assessment could reveal new insights into the pathophysiology of cardiovascular disease (CVD) or offer earlier detection and finer disease stratification, detailed in-vivo MiVED observation remains challenging due to a lack of suitable technologies. To address this gap, we hypothesized that accelerating ultra-wideband raster-scan optoacoustic mesoscopy (RSOM), i.e., fast RSOM (fRSOM), could resolve for the first time cutaneous MiVED features at single capillary resolution. We investigated whether we could record morphological features and dynamic responses during post-occlusive reactive hyperemia to achieve the most detailed observation of microvascular endothelial function to date. Our results show that using fRSOM on skin clearly measured the effects of smoking (N = 20) and atherosclerotic CVD (N = 20) on cutaneous endothelial function. For the first time, we found layer-specific effects, with smoking and CVD affecting the sub-papillary dermis differently than the reticular dermis; a finding not resolvable using “bulk” volumetric signals from laser Doppler flowmetry or tissue spectrometry. Interestingly, we observed no substantial structural changes in the microvasculature of smokers and volunteers with CVD, indicating that MiVED may be an earlier marker than morphology-based biomarkers typically assessed by histological studies. Our study introduces a non-invasive modality that enables the visualization and quantification of skin microvascular structure and function, bridging a technological gap and offering new insights into the effects of diseases on MiVED. This study potentially paves the way for fRSOM use as an early detection, diagnostic, or theranostic marker.

## Introduction

Microvascular endothelial dysfunction (MiVED) is implicated in a number of health conditions, such as hypertension, atherosclerotic cardiovascular disease (CVD), and diabetes^1–7^. There is mounting evidence that MiVED precedes macrovascular endothelial dysfunction (ED), potentially providing an earlier marker of disease development^8–10^. However, although the need for accurate assessment of MiVED, for example, in CVD, has been acknowledged^11^ and cutaneous microvasculature could serve as an accessible vascular bed to investigate MiVED for early detection and monitoring of diseases, clinical investigations continue to routinely assess macrovascular ED by means of ultrasonography of arteries during flow-mediated dilation (FMD) tests. This reliance on ultrasonography is due to a current lack of appropriate tools for MiVED assessment.

Peripheral arterial tonometry (PAT), near-infrared spectroscopy (NIRS), and various Doppler-based methods have been considered for non-invasive MiVED assessment, but each method has different limitations. PAT is a single-point method^12,13^ that records bulk blood flow and volume in tissues but not in microvasculature^12,14,15^. Due to strong photon scattering in tissue, NIRS methods also measure bulk signals from muscle perfusion and oxygenation during post-occlusive reactive hyperemia (PORH) tests without any possibility of resolving microvasculature^16,17^. Likewise, Laser Doppler flowmetry (LDF) and imaging (LDI) are highly affected by photon scattering and only provide bulk superficial measurements from skin volumes that are larger than 1 mm^3^ ^18–20^. To elaborate, flowmetry reports the average of individual capillary flows along different directions. This measurement does not reflect the actual condition of the microvasculature but instead only its macroscopic and mixed effect. Furthermore, at frame rates of ~1 min/frame, LDI is not suited for recording dynamic responses during PORH tests^21–23^. In addition, combined with the use of mathematical models that typically offer crude approximations to the tissue optical properties, averaging operations of Laser Doppler methods may also lead to poor reproducibility^18,23^. Laser speckle contrast imaging (LSCI) was more recently developed to resolve blood flow and perfusion based on speckle contrast^24^, but similarly to other optical methods, it is sensitive to photon scatter, does not resolve beyond a depth of about 300 µm^21,22^ and may suffer from spatial variability^25,26^.

Moving beyond bulk superficial measurements, dynamic optical coherence tomography (D-OCT) offers resolution reaching tens of micrometers and enables visualization of microvasculature^27–29^. D-OCT has been used to characterize changes in cutaneous microvascular function in subjects with diabetes compared to healthy volunteers^29^ and to assess the impact of age and blood pressure on dermal features during a reactive hyperemia process^30^. However, the temporal resolution of the method (about 60 to 90 s) only allowed the collection of images at two time points, once before and once after releasing arterial occlusion^30^, and as such was not suited to continuously track and quantify the fast dynamic changes occurring in the skin microvasculature during a PORH test. The slow acquisition is also prone to motion artifacts, i.e., observation between time points is affected even by slight movements of the subject between scans, leading to poor quality of the microvasculature function measurement^26,29,31^. In addition, projection artifacts in the axial direction compromise the ability of D-OCT to obtain high-quality cross-sectional images of the skin^31,32^ and reduce the overall D-OCT penetration depth to about 500 µm^27–29,31^. To address these limitations, Raster Scan Optoacoustic Mesoscopy (RSOM) has been introduced, offering leading performance in micro-vasculature imaging^33–35^. Implemented with ultra-wide bandwidth (UWB: 10–120 MHz), the method has enabled visualization of morphological micro-vasculature changes in dermatology^33,36,37^, at resolutions of up to 4.5 μm in the axial direction and 18.4 μm in lateral direction^33^. The high resolution achieved along all three dimensions allowed a unique characterization of inflammatory responses based on single capillary analytics without the use of contrast agents. RSOM has been already employed in human studies to quantify psoriasis burden and remission due to treatment^33,36^, visualize vasculature associated with melanoma formation^38^ or capture microvasculature complications of patients with diabetes^39^.

Aiming to radically improve the precision of MiVED assessment, we hypothesized that acceleration of optoacoustic mesoscopy would capitalize on the superior RSOM performance and enable the study of MiVED features at a single capillary level, i.e., achieving unprecedented ability and spatial and temporal resolution over any other method used today. The acceleration of RSOM was performed by two primary advances. The first advance was the use of ultrasound detectors with an opening through the center of the transducer’s active area to enable coaxial illumination and detection. For a set illumination energy per unit area dictated by safety limits of tissue exposure to light, coaxial illumination reduces the illumination area on skin and allows to increase laser repletion rate compared to previous schemes using illumination around the transducer, thus reducing the time required for signal acquisition^38,40^. The second advance was the introduction of novel scanning protocols, allowing dynamic control of the scan area, therefore enabling switching between grid scans to provide three-dimensional (3D) volumetric images and line scans to provide fast cross-sectional two-dimensional (2D) images.

Termed fast-RSOM (fRSOM), we established an accelerated RSOM method that was applied to examine whether functional microvasculature parameters could be resolved at a single capillary level and skin layer-dependent manner under a PORH test. Furthermore, we used fRSOM to investigate the so far unknown relationships between health conditions and single capillaries or depth-specific features. We show that fRSOM can resolve dynamics of individual cutaneous capillaries at 2 Hz temporal resolution at least, which is necessary for capturing changes during a PORH test. Based on this novel information and ability, we introduce three unique MiVED biomarkers, i.e., the maximum volume change (MVC), hyperemia ratio (HR), and time-to-peak (TP), at the single capillary level and as a function of capillary depth. The definition of such biomarkers was not possible before since no other method is able to retrieve such capillary metrics. While a significantly larger number of biomarkers can be extracted from the rich information data set collected by fRSOM, the selection of these three biomarkers allows an in-depth pilot understanding of the fRSOM’s unique features and their relationship to different conditions.

We have employed these unique biomarkers to study for the first time the effects of smoking and CVD on micro-endothelial function on a spatially dependent basis. Furthermore, the skin was used as a window to non-invasively assess the systemic effects of these conditions. We observed MiVED alterations in both the smoker and CVD groups and recorded previously undocumented impairment differences in shallow versus deeper dermal microvessels. In parallel, we observed no clear vascular morphology changes. The results are contrasted to results obtained using spectral LDF and partial blood volume and oxygen saturation measurements by white-light tissue spectrometry (WLS). We discuss how fRSOM imparts unprecedented detail when non-invasively assessing microvasculature function, and its potential to enable for the first time systematic or longitudinal cutaneous MiVED observations. This novel in-vivo assessment ability has been heralded in the literature as a necessary technology that could, in the future, enable early detection of disease progression, i.e., earlier than first observation of functional alterations in larger vessels based on technologies available today. fRSOM can also provide quantitative biomarkers required for personalized prevention programs or for following up on disease progression and interventions over long periods of time.

## Results

**Fig. 1:**
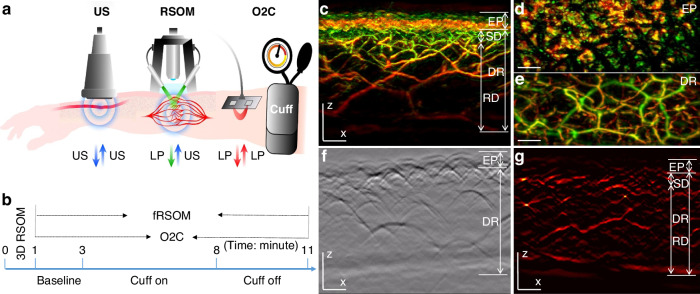
**fRSOM procedure to assess skin microvasculature endothelial function.** **a** Schematic illustration of assessment of skin microvascular endothelial function at the forearm by fRSOM and O2C, and macrovascular function assessed by ultrasound (US) during a PORH (post-occlusive reactive hyperemia) test (see Methods); O2C: oxygen to see, a commercial system including laser Doppler flowmetry (LDF) and White-Light Spectroscopy (WLS), simultaneously records blood flow, partial blood volume (rHb) and oxygen saturation (SO_2_), LP: laser pulse. **b** The timeline showing 3D-RSOM, fRSOM, and simultaneous LDF and WLS assessments during a post-occlusive reactive hyperemia (PORH) test (2 min baseline, 5 min after inflating the cuff (Cuff on) and 3 min after deflating the cuff (Cuff off)). **c** Cross-sectional image of the 3D-RSOM scan at the forearm of a healthy volunteer. Corresponding MIP images of the (**d**) epidermis (EP) and (**e**) dermis (DR) layers of (**c**) in the coronal direction. The 3D RSOM images are color-coded to represent the two reconstructed frequency bands (red: larger structures in the bandwidth of 10–40 MHz; green: smaller structures in the bandwidth of 40–120 MHz). The 3D RSOM volume was acquired in a region 4 mm (x-axis) × 2 mm (y-axis), and z is the depth axis. **f** Raw optoacoustic signals of one fRSOM line scan. The scan region of fRSOM is 4 mm (x-axis) × z (depth axis). **g** The reconstructed image corresponding to (**f**). The arrows show the upper subpapillary dermis (SD) layer and the lower reticular dermis (RD) layer—scale bar: 500 µm

In order to assess cutaneous MiVED, we conducted forearm PORH tests (see Methods, Fig. 1a, b) and examined whether fRSOM could directly visualize the dynamics of the microvasculature at a single capillary level from the surface of forearm skin in humans. Measurements were compared to LDF and WLS measurements (O2C^©^ system) to provide indirect indicators of microvascular perfusion. The PORH procedure (Fig. 1b) included three sets of measurements: 2 min of baseline, 5 min of “cuff on” (inflated cuff at a pressure of at least 40 mmHg above the systolic blood pressure of the subject), and 3 min of “cuff off” (deflated cuff). fRSOM obtained 3D volumetric scans (~4 × 2 × 1.5 mm^3^, Fig. 1c–e) in 1 min and 2D cross-sectional scans (~4 × 0.1 × 1.5 mm^3^; Fig. 1g) in 0.5 s that allowed examination of the response to PORH with high spatial or temporal detail. Due to the use of a standard scanner for 3D and 2D images, the position of the cross-sectional images obtained during PORH can be accurately referenced to the corresponding 3D fRSOM images. These two modes are exemplified in Fig. 1c–g. Cross-sectional images, shown as Maximum Intensity Projections (MIPs) of the 3D fRSOM scans, acquired at the forearm skin of a healthy volunteer (Fig. 1c), revealed details of the epidermis and dermis layers at a depth of about 1.5 mm at the illumination wavelength of 532 nm (see Supplementary Fig. S1). MIP images of the 3D volume along different projections depict superficial skin ridges (Fig. 1d), capillary loops and the vascular morphology of the vascular plexus (Fig. 1c, e). Inspection of the raw optoacoustic signals of the 3D fRSOM scan (Fig. 1f) provides a visual assessment of the acquired signal quality. Finally, the reconstructed 2D image of the fRSOM line scan (Fig. 1g) resolves skin epidermis and dermis layer structures that relate well with the 3D MIP image in Fig. 1c. The fRSOM line scan data during an entire PORH test are shown in supplementary Movies 1, 2.

**Fig. 2:**
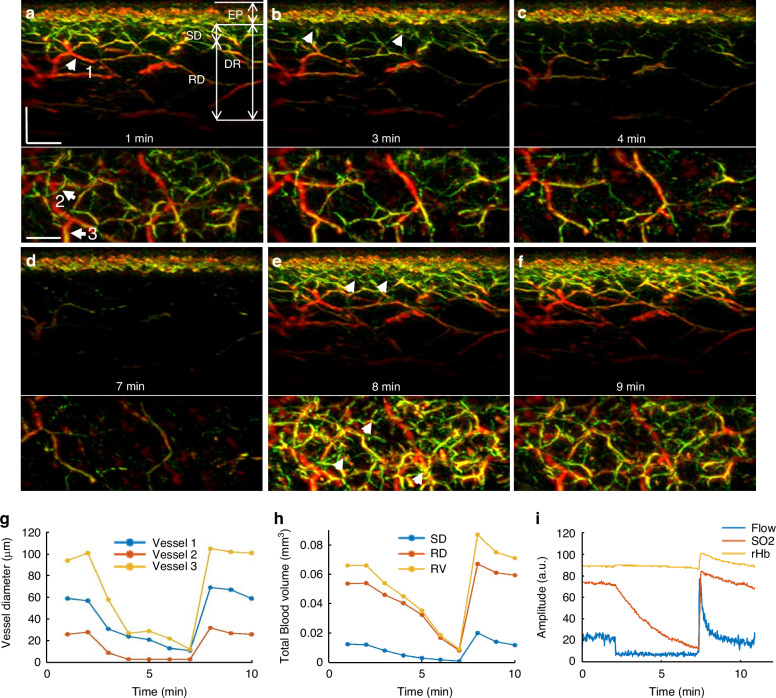
**3D-RSOM imaging of skin microvasculature hyperemia during PORH.** The skin vasculature at the forearm (4 × 2 mm^2^) of a healthy volunteer was measured by 3D-RSOM at every minute during the 10 min PORH test. **a**–**f** Cross-sectional images of 3D-RSOM volumes were acquired at six time points with corresponding MIP images of the dermis layer in the coronal direction below each cross-sectional image. The white arrows in (**e**) indicate vessels that were visualized for the first time during the reactive hyperemia process. **g** The diameter changes of three vessels (white arrows 1, 2, and 3 in a) are characterized as the FWHM (full width at half maximum) values during the PORH test. **h** Changes in the total blood volume in the subpapillary dermis (SD) layer, the reticular dermis (RD) layer, and the whole dermis vasculature (DV) were computed from the 10 RSOM images obtained during the PORH test. **i** LDF&WLS measurements, including the blood flow, oxygen saturation (SO_2_), and partial blood volume (rHb) during the PORH test. Scale bar: 500 µm

To offer a first look ever into the 3D hyperemia response of the skin microvasculature during a PORH test, we obtained ten 3D-RSOM images from the forearm of one healthy volunteer (female, 29 years old) at 1-min intervals (Fig. 2 and Supplementary Fig. S2). Cross-sectional 3D-RSOM images at six-time points and corresponding MIP images of the dermis (DR) vasculature in the coronal plane (Fig. 2a–f) clearly identified significant changes of skin microvasculature across skin depths during hyperemia (see also Supplementary Movies 3, 4 and images in Supplementary Fig. S2). 3D-RSOM images at the baseline (Fig. 2a) resolved rich vasculature in the skin. After initiating the cuff inflation during a PORH test, microvessels in the subpapillary dermis layer (SD, white arrows in Fig. 2b) became less visible (Fig. 2c) and virtually disappeared from the images after 7 min (Fig. 2d and Supplementary Fig. S2), while the melanin layer in the epidermis (EP), serving as a reference, remained constant. Likewise, the vessels in the deeper reticular dermis (RD) layer gradually became invisible (Fig. 2b–d), and the overall image intensity dropped significantly compared to the baseline image (Fig. 2a). After deflating the cuff, a strong hyperemia response was recorded: vascular features were fully recovered, and additional cutaneous vessels appeared (marked by five white arrows on Fig. 2e). The dilation of three vessels during hyperemia (marked by the white arrows labeled 1, 2, and 3 on Fig. 2a) was quantified by measuring the vessel diameter at every minute (Fig. 2g), showing the variability of response patterns of individual capillaries to pressure-induced stimuli depending on their size; larger vessels showed more prominent responses. For example, the mean diameter of vessel 1 was 58 µm at baseline and increased to 69 µm at peak hyperemic response (18% increment of the vessel diameter) after cuff deflation. The mean diameter of vessel 2 was 27 µm at baseline and dilated to 33 µm at peak hyperemia response (22% increment of the vessel diameter) after cuff deflation.

The average (total) hyperemia process was characterized based on changes observed in the total blood volume computed from 3D-RSOM images (see Methods), as shown in Fig. 2h, where the total blood volume of the subpapillary dermis, reticular dermis, and the whole dermal vasculature (DV including SD and RD layer together) were computed separately. The layer-dependent changes also demonstrated for the first time different responses to the PORH test, indicating that independent layer-specific biomarkers can be extracted from the skin and may contain additional information related to the condition studied. In particular, the response of the subpapillary dermis (SD) appears markedly weaker than the response of the reticular dermis (RD).

Overall, the analyses in Fig. 2 showed the first observations of capillary-specific and layer-specific changes in cutaneous micro-vessels at single-capillary resolution during the reactive hyperemia process. Thus, 3D-RSOM enables quantification of variability and depth-dependent responses not possible by other methods that only resolve bulk and possibly surface-weighted signals, i.e., the output of a single value per method (Fig. 2i).

**Fig. 3:**
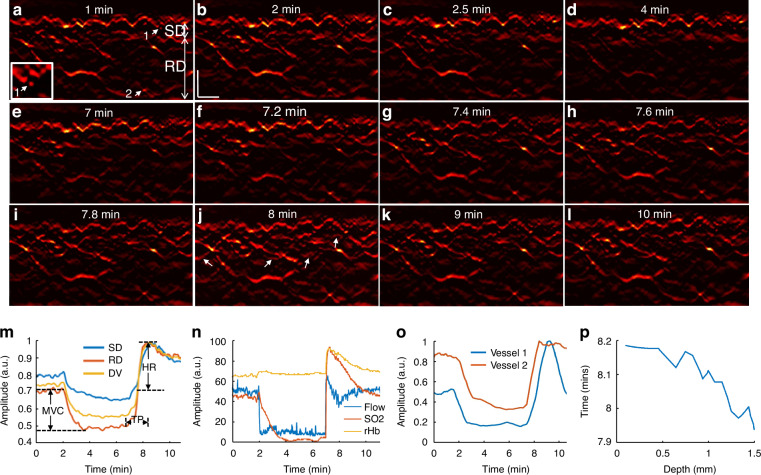
**fRSOM imaging to quantify skin microvasculature hyperemia during PORH test.** 12 fRSOM images at different time points during a PORH test are shown in (**a**–**l**). The white arrows in (**j**) indicate capillaries and dermal vessels that were not seen in baseline images and are first visualized upon release of the arterial occlusion. **m** Normalized vessel density changes in the subpapillary dermis (SD) layer, in the reticular dermis (RD) layer, and the whole dermis vasculature (DV) during the arterial occlusion process. **n** LDF and WLS measurements, including changes in the blood flow, oxygen saturation (SO_2_), and partial blood volume (rHb). **o** Normalized vessel intensity profiles of vessels 1 and 2 (labeled by the white arrows in (**a**) and the magnified inset). **p** Time points at peak hyperemia response after deflating the cuff in microvasculature located at various depths in the skin—scale bar: 500 µm

While the 3D mode of operation yields highly detailed volumetric insights into skin MiVED, each scan takes about one minute to scan a pattern of 4 × 2 mm^2^ on the skin area. Such temporal resolution is not well suited for detailed capturing of the dynamics of the hyperemia process, as seen in Fig. 2g, h. To overcome this limitation, fRSOM is able to scan faster in a different pattern over a 4 × 0.1 mm^2^ scan area, such that it can resolve cross-sectional skin images at up to two images per second, allowing capture of MiVED dynamics with up to 2 Hz temporal resolution. In fRSOM’s case, only capillaries on a single slice are captured (Fig. 3), but with finer dynamics (compare Fig. 3m to Fig. 2h). To exemplify the operation of the 2D mode, we show 12 fRSOM skin cross-sectional images (Fig. 3a–l) from a healthy volunteer (male, 34 years old) at different time points during the PORH test. The images resolve finer dynamic microvasculature changes in the subpapillary dermis (SD) and reticular dermis (RD) layers (see Supplementary Movie 5). Figure 3a–d shows skin images at different time points within the two-minute baseline period (Fig. 3a, b) and within the first two min after inflating the cuff (Fig. 3c, d). In complete agreement with the pilot 3D-RSOM measurements presented in Fig. 2, the dermal vasculature gradually disappeared while the intensity of the melanin layer in the epidermis layer, serving as a reference, remained constant. After deflating the cuff, a strong hyperemia response was observed over the first minute (Fig. 3e–j), both in the subpapillary dermis and reticular dermis layers with the appearance of previously unseen vessels as indicated by white arrows in Fig. 3j. Following the hyperemia phase, the microvasculature returns to its baseline appearance (Fig. 3k–i).

Image intensity profiles of the subpapillary dermis, reticular dermis, and whole dermal vasculature (Fig. 3m) allowed layer-dependent quantification of microvasculature density changes seen in Fig. 3a–l. Similarly to the observations done with the 3D images, different layers were shown to respond differently in the PORH challenge; these changes were captured with approximately 100 times better temporal resolution compared to Fig. 2. In contrast, each of the blood flow, oxygen saturation (SO2), and partial blood volume (rHb) tests resolved only a single surface-weighted value from the skin (Fig. 3n). Comparison of the fRSOM measurements to flow (or SO2) measurements revealed that the change of vessel density, as characterized by fRSOM, was slower than the perfusion changes recorded by LDF, indicating that the particular fRSOM measured vessel density signal discerns a complete restoration of the microvasculature system, contrasting with flow changes that overlook the influences of blood volume effects. Moreover, fRSOM was found to have significantly better inter-day repeatability and reproducibility compared to LDF and WLS (see Methods, Supplementary Figs. S3 and S4).

We further observed that analysis of two different micro-vessels (labeled 1 and 2 in Fig. 3a) showed that capillaries in the subpapillary dermis layer (arrow 1) responded earlier to the cuff occlusion compared to the larger vessels (arrow 2) seated deeper in the reticular dermis layer (Fig. 3o). However, the capillaries recovered in a delayed manner compared to the larger vessels. Figure 3p captures this observation for the entire skin volume sampled, confirming that dermal vessels deeper in the skin reach peak hyperemia ~15 sec faster than superficial vessels.

The dynamics recorded with high temporal resolution in Fig. 3m allowed the extraction of a number of biomarkers. To exemplify this potential and offer a quantitative assessment of endothelial function, we computed three parameters from fRSOM dynamic measurements, i.e., the maximum volume change (MVC), hyperemia ratio (HR), and the time-to-peak (TP) from the intensity profiles of fRSOM, as illustrated in Fig. 3m (see Methods for details). These biomarkers were computed in a layer-specific manner and were applied to assess MiVED in subjects with increased cardiovascular risk. 10 non-smoking volunteers (mean age 30.1 ± 2.3 years) and 10 age-matched smokers (mean age 33.5 ± 3.5 years) were measured during a 10-minute PORH test. LDF&WLS signals were simultaneously recorded for comparison.

**Fig. 4:**
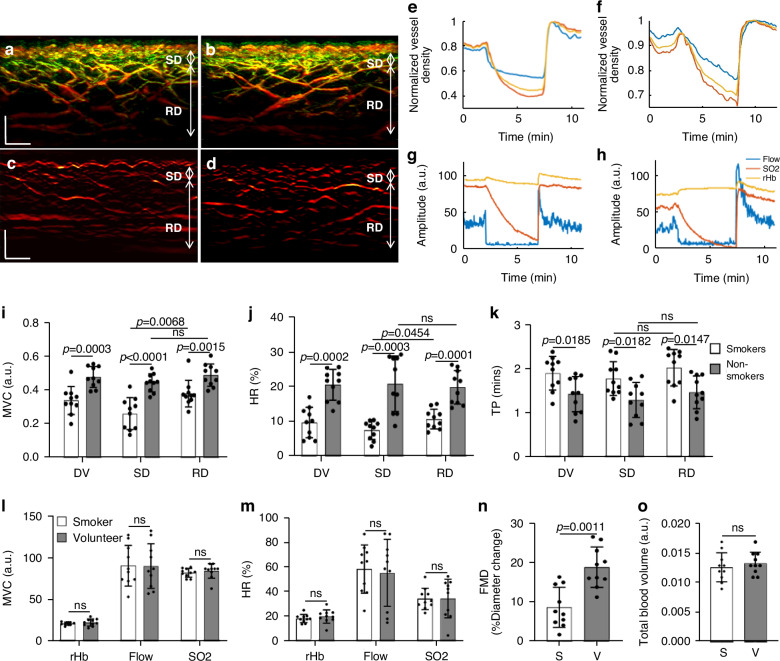
**Quantification and comparisons of skin microvasculature endothelial function between non-smokers and smokers.** Cross-sectional 3D-RSOM images of a male non-smoker (**a**) and a male smoker volunteer (**b**). **c**, **d** fRSOM images correspond to (**a**, **b**), respectively. **e**, **f** The image intensity profiles used to characterize endothelial function during the PORH test on a non-smoker and a smoker. **g**, **h** Signal profiles of the LDF and WLS measurements done on the same subjects, showing the blood flow, partial blood volume (rHb), and oxygen saturation (SO_2_) values. **i**–**k** Comparisons of the MVC (maximum volume change), HR (hyperemia ratio), and TP (time-to-peak) values in smoker versus non-smoker groups. Values were computed from readings taken in the microvasculature of the subpapillary dermis (SD) layer, the reticular dermis (RD) layer, and the whole dermis vasculature (DV). **l**, **m** Measurements of MVC and HR values calculated from the LDF&WLS measurements. **n** Macrovascular endothelial function assessed by ultrasound flow-mediated dilation during PORH test in non-smokers (V) and smokers (S). **o** Comparisons of the total blood volume in the dermal layer of non-smokers versus smokers. ns not significant. Scale bar: 500 µm

Figure 4a–d shows two cross-sectional MIP 3D-RSOM images of the skin at the forearm of a male non-smoker (Fig. 4a) and a male smoker (Fig. 4b), and two corresponding fRSOM images (Fig. 4c, d). The intensity profiles computed from the fRSOM signals during the PORH test for the non-smoker (Fig. 4e) and the smoker (Fig. 4f) showed good temporal agreement with LDF&WLS measurements (Fig. 4g, h). The MVC values (Fig. 4i) of the smoker vs non-smoker groups were 0.34 ± 0.082 (a.u.) and 0.48 ± 0.062 (a.u.) for the entire dermis vasculature (DV) layer, 0.26 ± 0.095 (a.u.) and 0.44 ± 0.054 (a.u.) in the subpapillary dermis (SD) layer and 0.38 ± 0.079 (a.u.) versus 0.49 ± 0.063 (a.u.) in the reticular dermis (RD) layer respectively. Unpaired t-tests and Wilcoxon signed-rank tests indicated significant differences (*P* = 0.0003 in DV, *P* < 0.0001 in SD, and *P* = 0.0015 in RD). We further observed that the MVC value (0.38 ± 0.095) of the subpapillary dermis layer was significantly lower than the MVC value (0.49 ± 0.063) of the reticular dermis layer in the smoker group (*P* = 0.0068). At the same time, there was no significant difference in the non-smoker group.

The hyperemia ratio (HR) values (Fig. 4j) were calculated to serve as a second biomarker with possible relevance to MiVED alterations. In the entire dermis vasculature layer, the smoker group exhibited HR values that were 9.54% ± 4.37%, i.e., significantly lower compared to 20.38 ± 4.43% in the non-smoker group. For smoker versus non-smoker groups, the HR values in the subpapillary dermis layer were 7.67% ± 3.52% versus 19.65 ± 4.84% and 10.52% ± 2.83% versus 20.62% ± 7.94% in the reticular dermis layer respectively. Unpaired t-tests and Wilcoxon signed-rank tests indicated significant differences (*P* = 0.0002 in DV, *P* < 0.0001 in SD, and *P* = 0.0036 in RD). Moreover, the HR values in the subpapillary dermis layer of the smoker group were significantly lower than the values in the reticular dermis layer (*P* = 0.0454), while there was no statistical difference in the non-smoking group.

The metric, speed of hyperemia response, measured as the time-to-peak (TP) after cuff deflation in Fig. 3m, also exhibited marked differences between the smoker and non-smoker groups (Fig. 4k). When comparing smokers versus non-smokers, the mean TP values were 1.89 ± 0.38 min compared to 1.42 ± 0.40 min in the entire dermis vasculature layer, 1.77 ± 0.38 min versus 1.29 ± 0.40 min in the subpapillary dermis layer, and 2.01 ± 0.41 min versus 1.46 ± 0.37 min in the reticular dermis layer. Unpaired t-tests and Wilcoxon signed-rank tests indicated significant differences (*P* = 0.0185 in DV, *P* = 0.0182 in SD, and *P* = 0.0147 in RD). There were no significant differences in TP values when the subpapillary dermis and reticular dermis layers were compared in the smoker or non-smoker groups.

Similar analysis of the MVC (Fig. 4l) and the hyperemia ratio values (Fig. 4m) derived from the LDF & WLS measurements did not demonstrate statistically significant differences between the smoker and non-smoker groups. A significant change (*P* = 0.0011) was nevertheless observed in the macrovascular endothelial function assessed by ultrasound during the PORH test when the smoker group was compared to the non-smoker volunteer group (Fig. 4n). Moreover, no marked differences between the smoker and non-smoker volunteer groups were observed in the total blood volume (Fig. 4o) computed from the 3D-RSOM images.

**Fig. 5:**
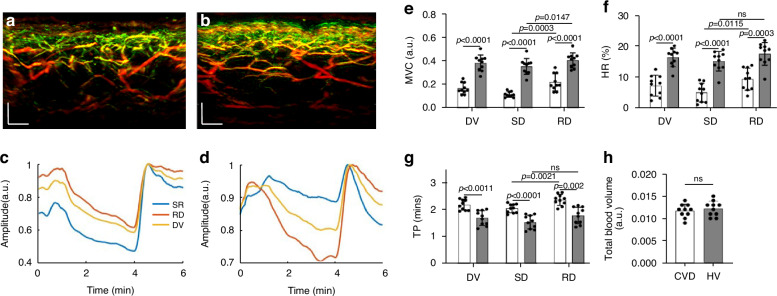
**Comparisons of skin microvasculature endothelial function between volunteers with and without CVD.** **a**, **b** Cross-sectional RSOM images of a healthy volunteer (**a**) and a CVD volunteer (**b**). **c**, **d** The image intensity profiles were used to characterize endothelial function during the PORH test on a non-CVD healthy volunteer and a CVD volunteer. **e**–**g** Comparisons of the MVC (maximum volume change), HR (hyperemia ratio), and TP (time-to-peak) values, computed from the fRSOM image intensity profiles, between the healthy volunteers (HV) and CVD volunteers. Values were calculated from readings taken in the microvasculature of the subpapillary dermis (SD) layer, the reticular dermis (RD) layer, and the whole dermis vasculature (DV). **h** Comparison of the total blood volume in the dermal layer between the healthy volunteers and CVD volunteers. ns not significant. Scale bar: 500 µm

To offer a pilot examination of MiVED biomarkers relevant to cardiovascular disease, we performed targeted measurements in volunteers with CVD and age-matched non-CVD volunteers. Ten volunteers with CVD were measured by fRSOM during a 6-minute PORH test and compared to 10 non-CVD volunteers (HV, see Methods). Figure 5a, b shows two cross-sectional MIP 3D-RSOM images of the skin at the forearm of a non-CVD volunteer (Fig. 5a) and a volunteer with CVD (Fig. 5b). The intensity profiles of a non-CVD healthy volunteer and CVD volunteer are shown in Fig. 5c, d (see Supplementary Fig. S6). The MVC values (Fig. 5e) of the CVD group versus the non-CVD group were 0.16 ± 0.048 versus 0.38 ± 0.068 in the entire dermis vasculature (DV) layer, 0.11 ± 0.024 versus 0.34 ± 0.050 in the subpapillary dermis (SD) layer and 0.21 ± 0.073 versus 0.40 ± 0.048 in the reticular dermis (RD) layer. Unpaired t-tests and Wilcoxon signed-rank tests indicated significant differences (*P* < 0.0001 in DV, *P* < 0.0001 in SD, and *P* < 0.0001 in RD). Furthermore, we found that the MVC value of the subpapillary dermis layer was significantly lower than the value of the reticular dermis layer in the CVD group (*P* = 0.0003) compared to the healthy volunteer group (*P* = 0.0147). The CVD group in comparison to the non-CVD group exhibited significantly lower hyperemia ratio (HR) values (Fig. 5f) of 7.13 ± 3.41% versus 16.38 ± 2.99% in the DV layer, 4.91% ± 3.04% versus 15.13 ± 3.21% in the subpapillary dermis layer, and 9.16% ± 3.45% versus 17.48% ± 3.69% in the reticular dermis layer. Unpaired t-tests and Wilcoxon signed-rank tests indicated significant differences (*P* < 0.0001 in DV, *P* < 0.0001 in SD, and *P* = 0.0003 in RD). Moreover, the hyperemia ratio values in the subpapillary dermis layer in the CVD group were significantly lower than the values in the reticular dermis layers (*P* = 0.0115), while there was no statistical difference in the non-CVD group. The mean time-to-peak values (Fig. 5g) of the CVD group compared to the non-CVD group were 2.18 ± 0.20 min versus 1.69 ± 0.27 min in the DV layer, 2.05 ± 0.16 min versus 1.53 ± 0.26 min in the subpapillary dermis layer, and 2.36 ± 0.20 min versus 1.78 ± 0.31 min in the reticular dermis layer. Unpaired t-tests and Wilcoxon signed-rank tests indicated significant differences (*P* = 0.0011 in DV, *P* = 0.0182 in SD, and *P* = 0.002 in RD). The time-to-peak values in the subpapillary dermis layer in the CVD group were significantly lower than the values in the reticular dermis layers (*P* = 0.0021), while there was no statistical difference in the non-CVD group. Similar to the smoking study, the total blood volume (Fig. 5h) related to the skin microvasculature structure showed no apparent differences between the CVD and the non-CVD groups.

## Discussion

In this study, we have pioneered a novel method for the precise assessment of cutaneous MiVED, allowing for the computation of biomarkers at an unprecedented level of granularity, down to the level of individual capillaries and specific skin layers. In stark contrast to other technologies considered for MiVED assessment, our fRSOM technique offers an unparalleled functional and temporal resolution. It achieves this without the need for averaging the contributions of multiple capillaries beneath the sensor. One of the distinguishing features of fRSOM is its ability to compute these biomarkers for each capillary and distinct skin layer. This capability has unveiled previously undiscovered aspects of MiVED that respond differently to various challenges and conditions, such as smoking or CVD, as demonstrated in our study. Crucially, fRSOM can provide this groundbreaking ability in a widely accessible manner, as it operates without the requirement for contrast agents or ionizing radiation. Additionally, it is portable and employs safe energy levels for the skin. As a result, we anticipate that fRSOM will have a wide range of applications in the near future, offering a multitude of layer-specific or capillary-specific MiVED biomarkers. These novel biomarkers hold the potential to yield fresh insights into the functional responses of the skin in relation to different conditions, the onset and long-term progression of diseases, disease staging, and the monitoring of interventions.

The importance of the fRSOM ability demonstrated herein can be better understood in the context of what is possible today by optical methods (e.g., LDF, LDI, and NIRS), which do not directly visualize skin microvasculature but instead provide an indirect indicator of microvascular reactivity^18,23^. Since these methods resolve bulk tissue responses, their measurements are affected by capillary and flow orientation^26^. Moreover, it was never feasible before to study depth-dependent or capillary-dependent micro-vascular responses. While fRSOM can also average measurements to a single value after post-processing, it offers a fundamentally different principle of operation and a new premise for understanding MiVED. The importance of this ability was demonstrated herein by studying the effects of smoking and CVD in a depth-dependent manner and showing conclusively for the first time, with statistical significance, that these conditions do indeed affect the human sub-papillary dermis and the reticular dermis differentially.

In this work, we focused on deriving three MiVED biomarkers (MVC, hyperemia ratio, and time-to-peak) associated with the hyperemic responses in different skin layers and found that MiVED responses vary with the skin layer. MVC demonstrated the closest relationship to smoking effects, and these effects were more pronounced in microvessels of the subpapillary dermis layer compared to vessels in the deep reticular dermis layer. However, there was no significant difference observed in the skin layers of the non-smoker group. Similar MVC findings were observed in the CVD study, whereby higher MiVED was found in the subpapillary dermis layer of the CVD group compared to the reticular dermis layer in the same group. Compared to the non-smoker group, we found that the MVC values of the subpapillary dermis layer in the non-CVD group were markedly lower compared to the reticular dermis layer. This could be explained by the significantly different ages between the two groups (30.1 ± 2.3 years vs. 63.6 ± 7.1 years) since aging is reported to alter microvascular endothelial function^2,41^. We observed similar trends in the HR values in both the smoker and CVD studies. In contrast, the time-to-peak (TP) values showed a weaker relation to the conditions examined compared to the MVC and hyperemia ratio values.

Several other observations were afforded by the new biomarkers possible with fRSOM. As observed in Fig. 3o, there is a lag in MiVED biomarkers in the different layers of the skin. Therefore, besides defining biomarkers based on the performance in each layer, a new class of biomarkers can also be introduced based on the relative onset in different skin layers or even between individual capillaries, e.g., capillaries of various sizes. Nevertheless, whereas functional biomarkers showed a statistically significant relationship to the conditions studied, morphological biomarkers did offer differentiation potential. This finding indicates that MiVED may precede morphological alterations and that it possibly offers more sensitive biomarkers for conditions like smoking or CVD. The majority of patients with CVD are ex-smokers, which is not surprising given that smoking is one of the most significant modifiable risk factors for CVD. Unfortunately, patient records often do not include detailed information about the duration of smoking cessation. As a result, we cannot fully rule out the impact of a patient’s previous smoking habits on MiVED. Future studies involving populations of CVD patients with no history of smoking would help to clarify this effect.

Currently, fRSOM scans a thin volume (4 mm × 0.1 mm) to achieve high temporal resolution for precisely capturing the fast dynamic changes during the occlusion measurements. To further enhance the signal quality and minimize the effects of tissue heterogeneity, fRSOM can be implemented to scan along multiple lines in a larger field of view of human skin while maintaining the scanning speed by using more sensitive detection and illumination configurations with a higher repetition rate of the laser source. In the future, more extensive scale and longitudinal or prospective studies are required to validate the relationship of fRSOM biomarkers to clinical impacts, i.e., the use of such parameters for early detection of disease onset or long-term monitoring of disease evolution and effects of intervention. For example, the value of MiVED assessment by fRSOM versus macrovascular ED assessment by the US in predicting CVD onset and progression could be determined by longitudinal assessment of high-risk individuals. Likewise, future investigations could utilize a larger number of biomarkers extracted from 2D or 3D fRSOM measurements over time to examine the combined prediction power of all biomarkers against different clinical questions and conditions within the spectrum of the metabolic syndrome. From a technology standpoint, multi-spectral fRSOM (employing illumination at many different light wavelengths) could be used to further investigate the oxygen saturation changes during PORH tests and explore the extraction of additional biomarkers that may relate to MiVED. Conversion to multi-spectral fRSOM could be achieved by utilizing a tunable laser or time- or frequency multiplexed wavelengths. The current fRSOM system is an affordable device, with costs similar to a typical ultrasound clinical system used for vascular imaging. It is also compact and, thus, highly portable, with a size similar to that of conventional ultrasound systems, making it ideal for use in clinical settings. For this current study, all examinations were performed in the vascular outpatient clinic in a standard examination room without the need for any spatial adjustments. Moreover, the costs and dimensions of the system are expected to decrease significantly in the future by using laser diode-based laser sources, as already demonstrated in an associated preliminary study. The future system is projected to have component costs in the low thousands of euro range, facilitating mass production and further reducing overall costs.

In summary, our study marks a groundbreaking milestone by establishing fRSOM as a method capable of yielding a diverse array of MiVED biomarkers, in stark contrast to current techniques that produce only a single output. Importantly, these biomarkers can provide insights at the level of individual capillaries and can be presented with respect to depth, skin layer, or specific capillary groupings, including their size distribution. Leveraging this distinctive capability of fRSOM to deliver novel biomarkers, we have uncovered that MiVED varies as a function of the skin layer for smoking and for CVD. The non-invasive and portable nature of this technology positions ideally for widespread dissemination and opens the exciting prospect of deploying a fresh set of biomarkers for precise MiVED assessment during routine clinical examinations of related diseases.

## Materials and methods

### Study design

All participants signed an informed consent before voluntary inclusion in the study. Studies were approved by the local ethics committee of the Technical University of Munich and are registered with the German Registry for Clinical Studies (DRKS00037748 and DRKS00037749). For the first series of measurements taken to explore the possible changes in MiVED in subjects with high cardiovascular risk, we included n_1_ = 10 non-smokers/healthy volunteers (6 males, 4 females, mean age 30.1 ± 2.3 years) and n_2_ = 10 smokers (7 males, 3 females, mean age 33.5 ± 3.5 years, mean cigarettes per day 8.6 ± 3.4, smoking history of 16 ± 3.9 years). All volunteers (non-smokers and smokers) were free of other cardiovascular risk factors and not taking any regular medication. For the second series of experiments aimed at measuring alterations of MiVED in subjects with overt CVD, we included another n_3_ = 10 healthy volunteers (5 males, 5 females, mean age 63.6 ± 7.1 years), and n_4_ = 10 volunteers with previously diagnosed CVD (9 males, 1 female, mean age 65.4 ± 9.9 years). Detailed information on the four groups is shown in Supplementary Tables 1–3. All participants gave written informed consent before the measurement. RSOM data quality was evaluated based on our previously developed RSOM quality evaluation approach and low-quality data was excluded^42^. Significantly, higher melanin concentrations in skin could decrease the penetration depth of our imaging system. The scanned regions from participants with strong melanin were excluded to minimize the influence of the melanin.

### MiVED tests

All tests were performed in a quiet room at normal temperature (≈24°C). Subjects were asked to lie in a supine position with the ventral side of the dominant distal forearm easily accessible. A blood pressure cuff of appropriate size was placed around the upper arm and remained there for the duration of the PORH test. Before tests, the subjects’ blood pressures were measured to confirm that all were normotensive (110–120/75–80 mmHg). For the first set of MiVED measurements (healthy volunteers/ non-smokers and smokers), a single 3D-RSOM scan was completed first. Next, periodic fRSOM imaging was conducted for 10 min, including 2 min baseline measurement (deflated cuff), 5 min arterial occlusion measurement (inflated cuff at a pressure of at least 40 mmHg more than the systolic blood pressure of the subject), and 3 min rest measurement (deflated cuff). For volunteer comfort, in the second set of measurements (CVD group and age-matched healthy volunteers), we followed a shorter 6 min protocol, including 1 min baseline measurement (deflated cuff), 3 min arterial occlusion measurement (inflated cuff at a pressure of at least 40 mmHg more than the systolic blood pressure of the subject) and 2 min rest measurement (deflated cuff).

### fRSOM imaging system and O2C validation

In the present study, we employed a custom-made portable RSOM imaging system featuring a transducer with a central frequency of 50 MHz (Fig. 1a), which has been described in detail in our previous studies^33,38,43^. An optically and acoustically transparent plastic membrane was affixed to the volunteer’s skin using surgical tape. The scanning head containing the laser output and an ultrasound transducer was brought close to the membrane in order to position the focal point of the transducer slightly above the skin surface and thereby maximize the signal detection for the tomographic reconstruction as shown in Fig. 1. An Onda laser (Bright Solutions, Italy) with dimensions of 19 × 10 × 9 cm^3^ was used to provide light at a wavelength of 532 nm. The repetition rate of the laser was 500 Hz, yielding an optical fluence of 3.75 µJ/mm^2^, which is under the safety limit^33^. For 3D-RSOM imaging, a field-of-view of 4 mm × 2 mm was scanned with a step size of 15 µm in the fast axis and 15 µm in the slow axis. The total scanning time of one 3D-RSOM measurement took about 60 s. After the 3D-RSOM scan, RSOM was switched to line scan mode for carrying out fRSOM imaging by moving the scanning head back-forth along the middle line (4 mm long) in the defined field-of-view of the 3D scan at a 500 Hz repetition rate to yield one frame per second during the 10 min PORH test. For each measurement, the RSOM scanning head was positioned at a distance about 5 cm from the wrist, while the LDF&WLS probe was fixed nearby, as illustrated in Fig. 1. A clinically used pneumatic cuff was placed at the level of the upper arm (i.e., distal to the site of brachial artery measurement) and controlled by an experienced operator. A commercial lightguide tissue spectrophotometry system^44^ that combines LDF and WLS probes was used to simultaneously record the blood flow, partial blood volume, and oxygen saturation during the occlusion-induced hyperemia for comparison with fRSOM measurements. For the macrovascular endothelial function assessment, a traditional ultrasound probe for arterial diameter measurements was placed over the distal radial artery (cross-sectional view) for the whole duration of the PORH test. An experienced operator manually controlled the cuff. fRSOM, LDF&WLS, and ultrasound recordings were fully synchronized throughout each test.

### Image reconstruction for 3D/fRSOM imaging

A motion correction algorithm was first applied to eliminate motion artifacts in the RSOM data^45^. For 3D-RSOM image reconstruction, the acquired RSOM signals for the 10–120 MHz bandwidth were resolved into two frequency bands: 10–40 MHz (low) and 40–120 MHz (high). Signals of the two frequency bands were independently reconstructed and combined to produce a final image as described in previous work^33^. The reconstruction time for each band took about 5 min, with the voxel size of the reconstruction grid being 12 µm × 12 µm × 3 µm. Finally, RSOM images were rendered by taking the maximum intensity projections of the reconstructed images along the slow axis or the depth direction, as shown in Fig. 1. For fRSOM image reconstruction, the acquired RSOM signals were filtered at a bandwidth of 10–120 MHz. Since the ultrasound detector used in RSOM has a diameter of 3 mm and a detection aperture of 60 degrees, optoacoustic signals of the fRSOM scan were integrated into thin volumes (4 mm × 0.1 mm × 2 mm). Thus, 3D reconstructions based on a beam-forming algorithm were used to generate a three-dimensional image stack (4 mm × 0.1 mm). The final reconstructed image was rendered by taking the maximum intensity projections along the slow axis, as shown in Fig. 1. In the beamforming algorithm, the projected signals are weighed by the simulated sensitivity fields of the ultrasound transducer to mitigate the out-of-plane artifacts.

### Biomarker computation to quantify endothelial function

The endothelial function of skin microvasculature was quantified based on the fRSOM image intensity changes during the occlusion measurements, while the signals of the blood flow, partial blood volume (rHb), and oxygen saturation (SO_2_) were directly read out from the commercial LDF&WLS system. The surfaces of 3D/fRSOM images were first flattened. Then, the epidermal and dermis layers were separated into two parts based on an in-house automatic segmentation algorithm. The mean values of the RSOM image intensities from the subpapillary dermis layer, reticular dermis layer, and the whole skin depths were computed separately to characterize the different responses to cuff pressure (Figs. 2h and 3m). As illustrated in Fig. 3m; several parameters were computed based on the profiles of fRSOM image intensity changes: mean values of baseline (MVB, 0–2 min), the mean values of signals when inflating cuff (MVIC, 2–7 min), the peak intensity value (PIV) and corresponding time point after deflating cuff. Three biomarkers were computed to quantify endothelial function, including the maximum volume change, the hyperemia ratio, and the time-to-peak intensity. The maximum volume change (MVC) was calculated as the difference between the MVB and MVIC values (MVC = MVB-MVIC). The hyperemia ratio (HR) was calculated as the ratio: (PIV- MVB)/PIV (%). The time-to-peak intensity (TP) relating to the hyperemia response speed was calculated as the time difference from the cuff release (end of 7 min) to the time of the peak intensity value. In addition, LDF&WLS signals were used to compute the three biomarkers in comparison with fRSOM.

### Statistics

All metrics were displayed with mean values and standard deviations as error bars. To assess the significance of the statistical differences for the metrics between non-smoker volunteers and smokers and age-matched healthy volunteers and volunteers with CVD, we performed parametric tests (unpaired t-test) for normally distributed data; otherwise, nonparametric tests (Wilcoxon signed-rank test) were applied.

### Repeatability and reproducibility test

We performed tests to evaluate the repeatability and reproducibility of fRSOM for endothelial function characterization while comparing it with LDF&WLS. The skin endothelial function of three healthy non-smoking young volunteers (2 females and 1 male, average age 29 ± 2 years old) were measured repeatedly by fRSOM imaging and LDF&WLS during PORH tests conducted on three consecutive days. Similar areas of the forearms of the three volunteers were measured every day by the same operator with similar environmental conditions. The image intensity profiles of fRSOM and signal profiles of LDF&WLS are shown in supplementary Fig. S4. We found that the variations of fRSOM measurements were much less than the profiles of LDF&WLS measurements. We further computed Pearson’s coefficient values and root mean square deviation (RMSD) values to characterize the inter-day repeatability and reproducibility, which reveals that the fRSOM signals showed much better inter-day repeatability and reproducibility. In addition, all signal profiles of fRSOM and LDF&WLS (including flow, SO_2,_ and rHb) in the non-smoker and smoker groups were normalized and are shown in supplementary Fig. S5. We also observed that the variations in the fRSOM signals were much less compared to the LDF&WLS signals.

## Supplementary information

Supplementary information

Supplementary movie 1

Supplementary movie 2

Supplementary movie 3

Supplementary movie 4

Supplementary movie 5

## Data Availability

The raw optoacoustic imaging data of smokers, non-smoking healthy volunteers, volunteers with CVD, and age-matched healthy volunteers that support the findings of this study are available from the corresponding author upon request after permission is obtained from the responsible authorities and the Ethics Committee of TUM University Hospital, Hospital Rechts der Isar, Technical University of Munich, Munich, Germany. Source data are provided in this paper. The image reconstruction and processing algorithms are described in detail in Methods and the cited reference. The image reconstruction code is shared in this public repository: 10.5281/zenodo.6466446.
