# Supplementary material for: Single-capillary endothelial dysfunction resolved by optoacoustic mesoscopy
Source: Light Sci Appl. 2026 Jan 3;15:37. doi: 10.1038/s41377-025-02103-6 (PMC12764958; doi:10.1038/s41377-025-02103-6)
Supplement: Supplementary file 1 — Supplementary information [file 41377_2025_2103_MOESM1_ESM.pdf]

Supplementary Information for Single-Capillary Endothelial Dysfunction resolved by Optoacoustic Mesoscopy

We have computed the image depths of all subjects in our study. The variations are quite low as shown in supplementary Fig. S1. In addition, our method assesses skin features that change longitudinally, which allows us to measure and track the same skin features. The possible variations will not affect our results.

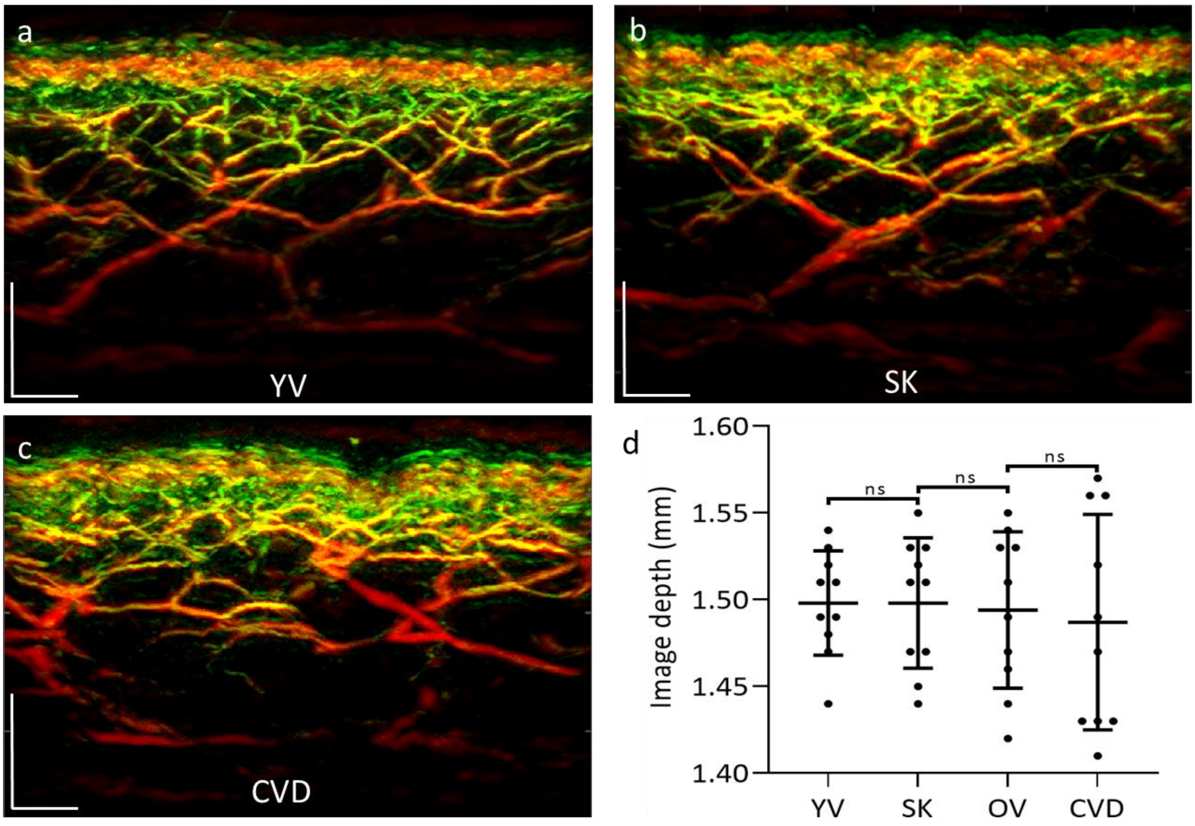

Supplementary Fig. S1. Assessment of the image depth among all objects in our study. In the smoker study, there are 10 young non-smoking volunteers (YV) and 10 smokers (SK). In the patient study, there are 10 old healthy volunteer (OV) and 10 patients with CVD. a-c, cross-sectional RSOM images of a young non-smoking volunteer (a), a smoker (b) and a patient with CVD (c). d. the depths of each group. The depth of YV group is 1.498 ± 0.030 mm, the depth of SK group is 1.497 ± 0.037 mm, the depth of OV group is 1.491 ± 0.051 mm, and the depth of CVD group is 1.487 ± 0.062 mm. There is no statistical difference among the groups. Scale bar: 500 µm, ns: not significant.

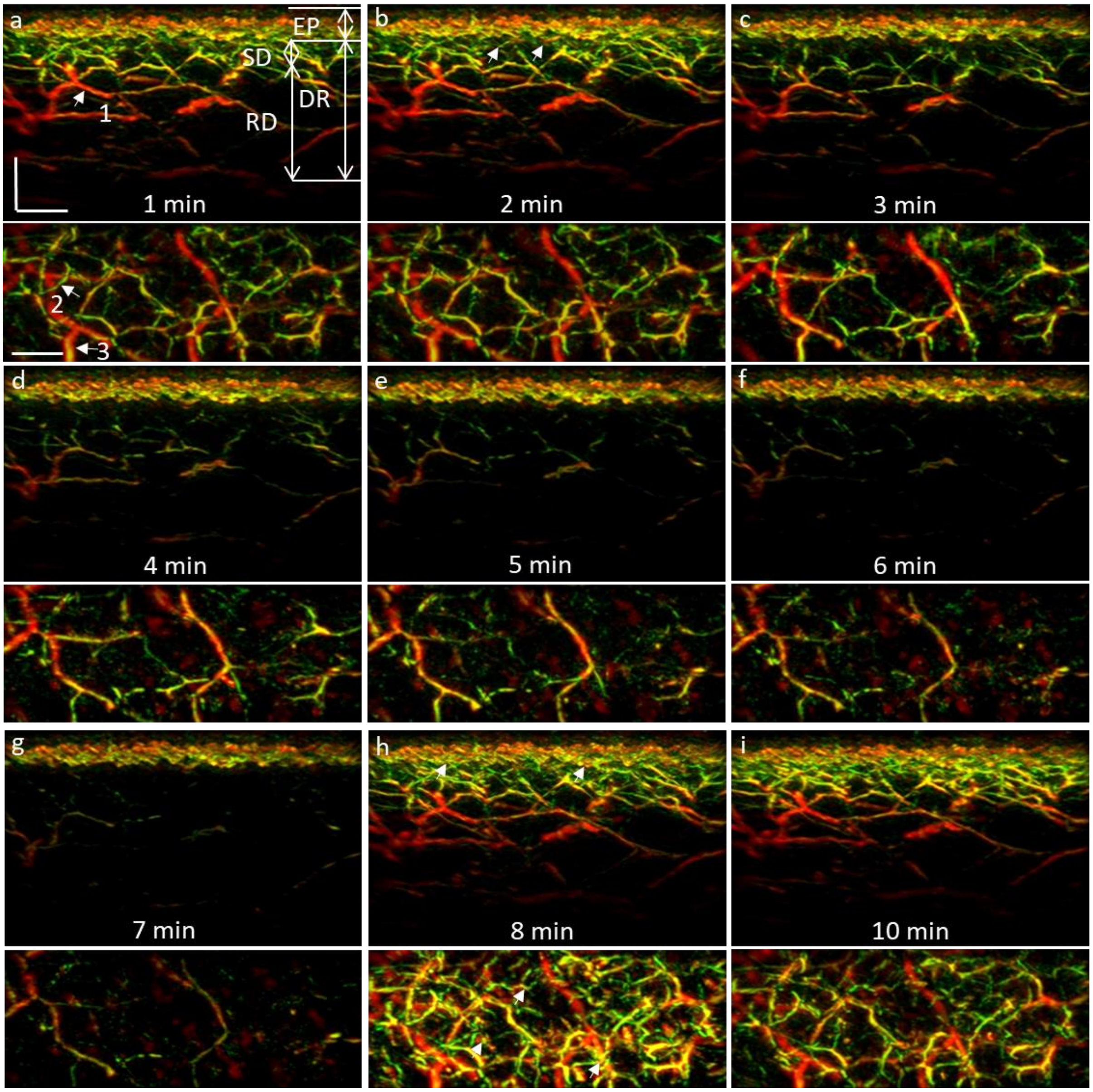

Supplementary Fig. S2. 3D-RSOM imaging of skin microvasculature hyperaemia during PORH. The skin vasculature at the forearm (4×2 mm2) of a healthy volunteer was measured by 3D-RSOM at every minute. Cross-sectional images of 3D RSOM volumes acquired at nine time points and corresponding MIP images of the dermis layer in the coronal direction (below each cross-section image) are shown in a-f. The white arrows in (h) indicate vessels that were visualized for the first time during the reactive hyperaemia process. Scale bar: 500 µm.

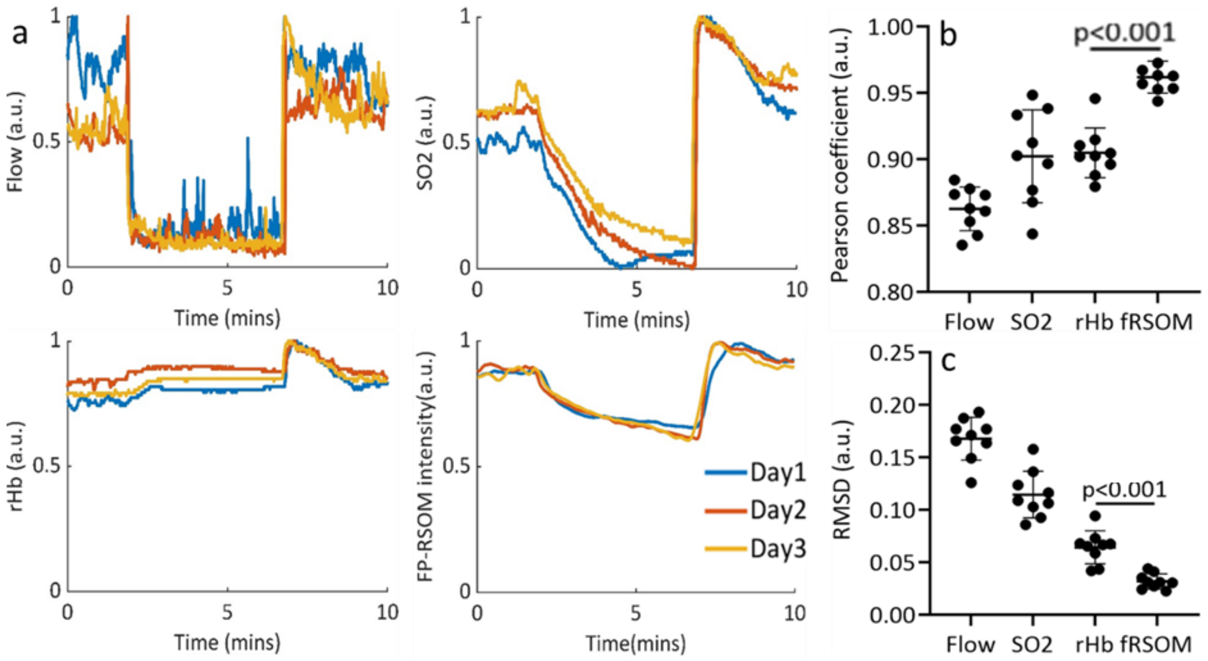

Supplementary Fig. S3. Repeatability and reproducibility comparisons among fRSOM, laser Doppler and tissue spectroscopy. a. the flow, SO2, rHb and fRSOM signals from a same healthy volunteer (female) measured on three consecutive days by commercial O2C and fRSOM simultaneously. The inter-day repeatability was characterized by computing Pearson's coefficient values and root mean square deviation (RMSD) values of each signal among the three days (day 1 vs. day 2, day 1 vs. day 3 and day 2 vs. day 3). The higher value of Pearson's coefficient means the better similarity between the signals while the smaller value of RMSD means the less difference between the signals. The mean Pearson's coefficient of the flow, SO2 and rHb are 0.83, 0.91, 0.92 while the value of fRSOM is 0.97. The RMSD values of the flow, SO2 and rHb are 0.15, 0,11, 0,05 while the value of fRSOM is 0.03. Both parameters demonstrate that the fRSOM signals show much better inter-day repeatability. To characterize the signals variations for the reproducibility test, we measured three healthy volunteers on the three consecutive days and computed the Pearson's coefficient and RMSD values as shown in b and c. It can be noted that the fRSOM has significantly higher Pearson's coefficient values and smaller RMSD values compared to the values of flow, SO2 and rHb, showing better reproducibility.

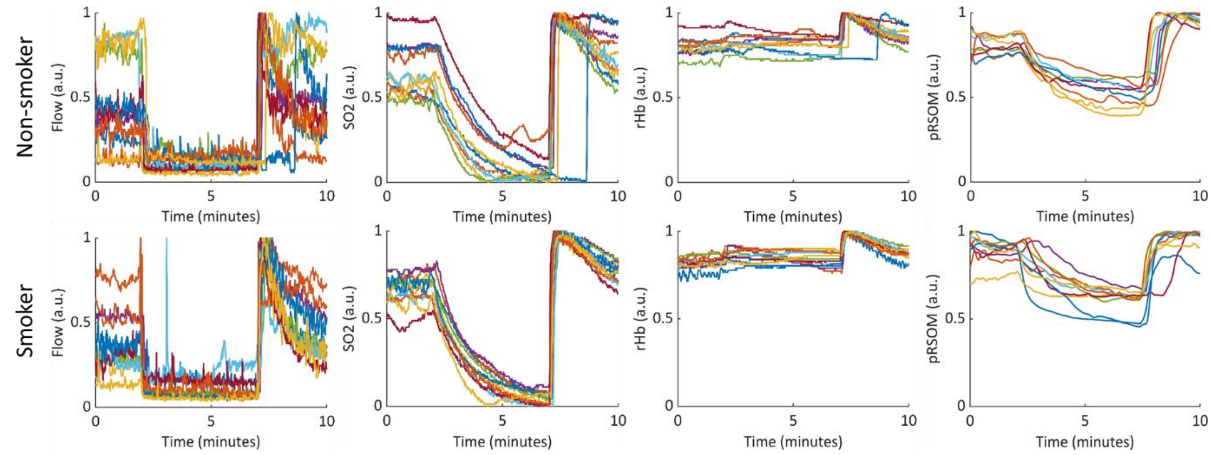

Supplementary Fig. S4. All signal profiles of the flow, SO2, rHb and fRSOM in the non-smoker healthy volunteer (n=10) and smoker (n=10) groups, related to Fig. 4. It can be noted that the signals of flow, SO2 and rHb show significant variations compared to fRSOM signals.

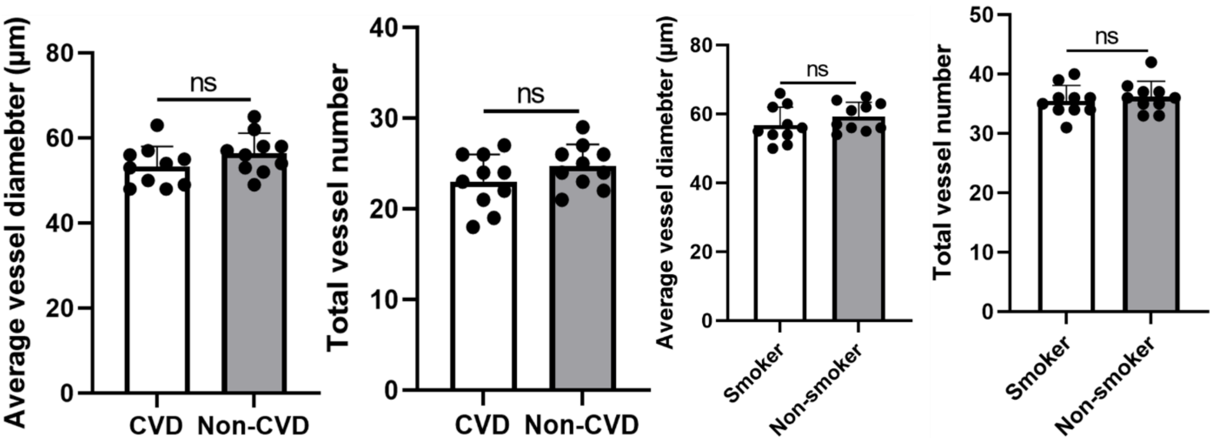

Supplementary Fig. S5. The average vessel diameter and the total vessel number comparisons between CVD vs. non-CVD and smoker vs. non-smoker groups, ns: no significance.

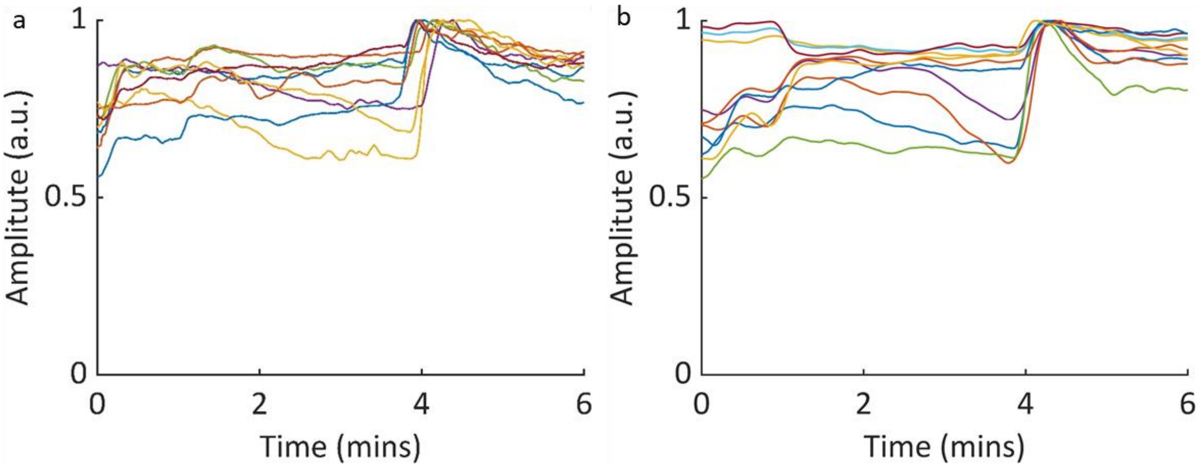

Supplementary Fig. S6. All signal profiles of fRSOM in the CVD patients (a) and healthy volunteers (b) groups related to Fig. 5.

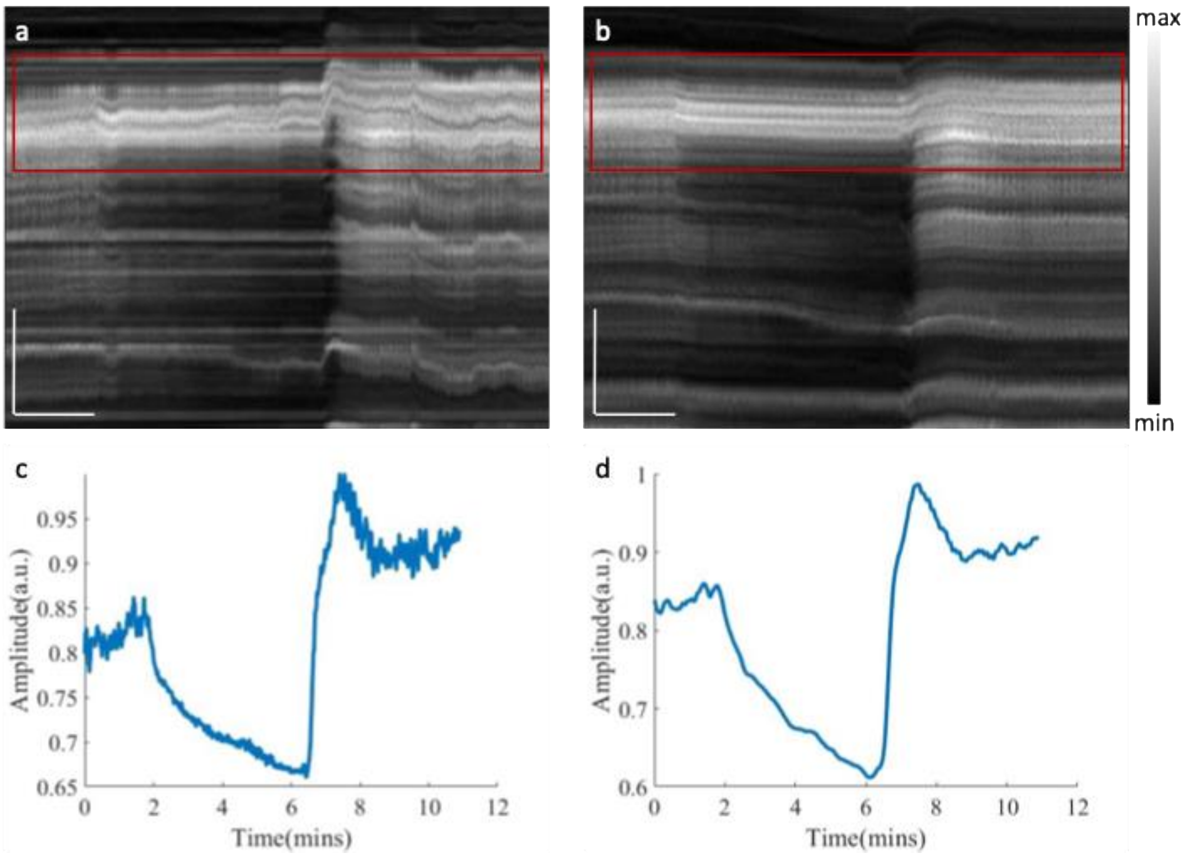

Supplementary Fig. S7. Motion correction for fRSOM imaging. (a) and (b): The maximum intensity signals of each B-plan fRSOM image during the occlusion process before and after applying our motion correction approach (x-axis corresponds to time from 0 – 12 mins). The red rectangles roughly indicate the positions of the peak signals in each B-plan fRSOM image. Motions artifacts that are prominent in (a) are corrected as shown in (b). (c) and (d): The corresponding intensity changes profiles during the same occlusion process. Signals variations in (d) are lower compared to the original profile (c). Scale bar: 500 µm.

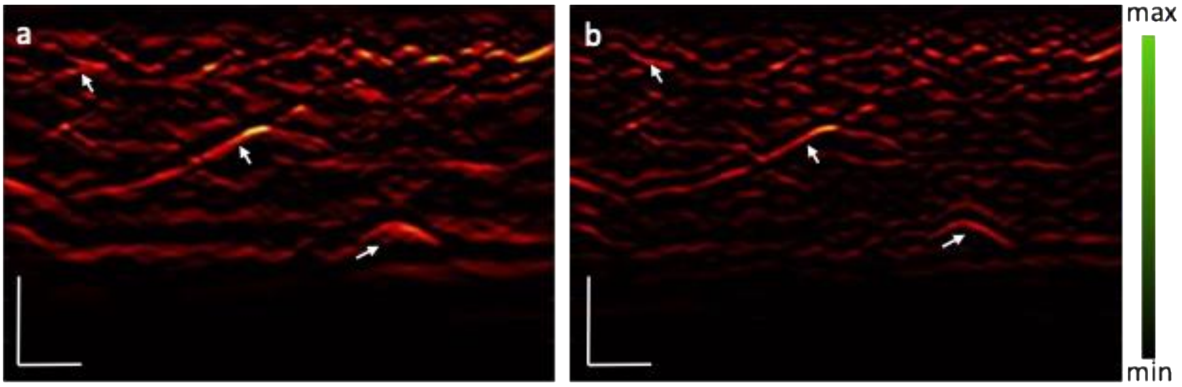

Supplementary Fig. S8. 3D fRSOM image reconstruction. (a) and (b): Reconstructed fRSOM images using 2D and 3D beamforming reconstruction algorithms, correspondingly. The 3D reconstruction algorithm is weighted by the detection sensitivity of the ultrasound transducer, which can minimize the effect of out-of-plane signals. As indicated by the white arrows, the visualized vascular features are more converged in the 3D reconstructed image (b) compared to the 2D reconstructed image (a). Scale bar: 500 μm.

Supplementary Table 1 Characteristics of study participants

	Smoker (n=10)	Non-smoker volunteer (n=10)	*P* value (smoker vs. non-smoker)	Patient with cardiovascular disease (n=10, CVD)	Healthy volunteer (n=10)	*P* value (CVD vs. healthy volunteers)
Age (years)	33.5 ± 3.5	30.1 ± 2.3	ns	65.4 ± 9.9	63.6 ± 7.1	ns
Gender (male/ Female)	7/3	6/4	na	9/1	5/5	na
BMI	24 ± 2.6	24.3 ± 1.6	ns	25.9 ± 1.1	24.7 ± 2.4	ns
Smoking duration (years)	16 ± 3.9	na	na	na	na	na
Smoking frequency (per cigarette/day)	8.6 ± 3.4	na	na	na	na	na
Exercise frequency (time per week)	1 ± 1.7	na	na	na	na	na

Note: na, not applicable; ns, not significant.

Supplementary Table 2 Detail information of 10 CVD patients

Patient number	BMI	Blood pressure	Smoking	Hypertension	Diabetes	CVD
01	24.5	110/85 mmHg	ex-smoker	yes	No	BAA, KHK
02	26.6	120/90 mmHg	ex-smoker	yes	Yes	PAD, KHK
03	27.2	110/85 mmHg	ex-smoker	yes	No	PAD
04	25.5	110/90 mmHg	no	yes	Yes	CAR, PAD, BAA, KHK
05	27	120/80 mmHg	ex-smoker	yes	Yes	CAR, PAD, KHK
06	25.8	120/70 mmHg	ex-smoker	yes	No	CAR, PAD, KHK
07	26.2	120/80 mmHg	ex-smoker	yes	No	CAR, PAD, KHK
08	24.2	110/70 mmHg	ex-smoker	no	No	PAD
09	26.3	130/80 mmHg	ex-smoker	yes	No	CAR
10	28	110/70 mmHg	no	yes	Yes	PAD, KHK

Note: KHK, coronary heart disease; BAA, aortic aneurysm; PAD, peripheral arterial disease; CAR, carotid atherosclerosis; CVD: Cardiovascular disease.

Our inclusion criteria are as follows:

- For the CVD group: Participants diagnosed with atherosclerotic cardiovascular diseases (coronary heart disease, carotid atheromatosis, and peripheral arterial occlusive disease), regardless of age or gender.
- For the non-CVD groups: Age- and gender-matched healthy subjects without any indication of cardiovascular disease.

Our exclusion criteria are as follows:

- Refusal to participate in the examination.
- Pregnancy.
- Strongly pigmented skin (leads to significant attenuation of the RSOM signal due to the presence of melanin in the skin).
- Documented or suspected light allergy, light intolerance, contact dermatitis, or particularly sensitive skin.
- Age under 18 years.
- Open or infected wounds at the measurement sites.

Regarding the skin: Most of the patients visiting our clinic where examinations took place are of North European origin, and their skin tones reflect these ethnic characteristics. For this study, all subjects included were of North European origin with the corresponding skin tones.

Supplementary Table 3 Detail information of 10 non-CVD patients

Patient number	BMI	Blood pressure	Smoking	Hypertension	Diabetes
01	23.5	110/70 mmHg	no	yes	no
02	25	125/80 mmHg	no	no	no
03	26	110/70 mmHg	no	no	no
04	21.6	117/70 mmHg	no	no	no
05	27.8	120/80 mmHg	no	no	no
06	29.8	125/85 mmHg	no	no	no
07	27.4	122/78 mmHg	no	no	no
08	27.8	128/80 mmHg	no	no	no
09	23.9	120/70 mmHg	no	no	no
10	26.4	124/78 mmHg	no	no	no

Note: KHK, coronary heart disease; BAA, aortic aneurysm; PAD, peripheral arterial disease; CAR, carotid atherosclerosis; CVD: Cardiovascular disease.

To understand the effects of the patient parameters on the fRSOM features between the CVD and non-CVD groups, we computed the Spearman correlation value between Age/BMI/Blood pressure with the fRSOM feature MVC (maximum volume change) and found no significant correlation as shown in Supplementary Table 4. We further applied multivariate logistic regression analysis to estimate the significance of MVC between the CVD and non-CVD groups including variables of age/BMI/Blood pressure, which showed no significant association between the fRSOM biomarker and patient parameters.

Supplementary Table 4. Correlation and multivariate logistic regression analysis between the fRSOM feature MVC (maximum volume change) and patient parameters

	Correlation*		Multivariate**	
	r	*p*	*β*	*p*
Age (years)	-0.07	0.51	0.92	0.0007
BMI	-0.05	0.47	0.86	0.0008
Blood pressure	-0.13	0.41	0.83	0.0009

Note: *The Spearman correlation value was calculated between MVC and the various patient parameter and no significant correlations were found; **Multivariate logistic regression analysis was applied to compute the significance of fRSOM biomarker MVC between the CVD and non-CVD groups including the variables of age, BMI and Blood pressure, which still show significant differences between the CVD and non-CVD groups. na: Not applicable. BMI: Body mass index, HbA1c: Glycated hemoglobin.

Supplementary Table 5. Correlation analysis between the average vessel diameter and the fRSOM features: the maximum volume change (MVC), hyperemia ratio (HR), and the time-to-peak (TP).

	Correlation*	
	r	*p*
MVC	-0.11	0.44
HR	-0.16	0.39
TP	-0.08	0.49

*The Spearman correlation value was calculated between the average vessel diameter and the three fRSOM features (MVC, HR and TP) and no significant correlations were found.

Supplementary Table 6. Non-invasive methods to assess the microvasculature endothelial function

Techniques	Principle	Pros	Cons	References
Near-infrared spectroscopy	Spectroscopic method that uses the near-infrared region of the electromagnetic spectrum	Simultaneously measures muscle perfusion and oxygenation, high temporal resolution	Bulk assessment of tissue, inaccuracy in the estimation of tissue optical properties, no depth information and no resolution to image microvasculature	Kragelj et.al, 2001[1]
Laser Doppler flowmetry	Based on the Doppler shift imparted by the moving red blood cells to the probing light	High sensitivity at detecting and quantifying relative changes in skin blood flow, high temporal resolution	Bulk assessment of tissue, poor reproducibility, no depth information and no resolution to image microvasculature	Varghese et.al, 2009[2]
Laser Doppler imaging	Based on the Doppler shift imparted by the moving red blood cells to the probing light	High sensitivity at detecting and quantifying relative changes in skin blood flow, good reproducibility	Low temporal resolution, no depth information and no resolution to image microvasculature, low temporal resolution	Newton et.al, 2001[3]
Laser speckle contrast imaging	Speckle contrast analysis that provides an index of blood flow	Image large area, high spatial resolution and good reproducibility	No depth information and no resolution to image microvasculature, shallow penetration depth (about 300 µm)	Barcelos et.al, 2017[4]
Optical coherence tomography angiography	Employ motion contrast imaging to high-resolution volumetric blood flow information generating angiographic images	High spatial resolution to image microvasculature	Current implementations take about 3 min to acquire volume, shallow penetration depth (about 500 µm)	Evers et.al, 2021[5]
fRSOM	Optoacoustic mesoscopy imaging	High spatial and temporal resolution to image microvasculature, whole skin depth penetration (up to few millimeters), good reproducibility	Relatively small field of view	

Supplementary information accompanies the manuscript on the Light: Science & Applications website (http://www.nature.com/lsa)

References

1. Kragelj, R., Jarm, T., Erjavec, T., Presern-Strukelj, M. & Miklavcic, D. Parameters of postocclusive reactive hyperemia measured by near infrared spectroscopy in patients with peripheral vascular disease and in healthy volunteers. *Ann Biomed Eng* **29**, 311-320 (2001).
2. Rajan, V., Varghese, B., van Leeuwen, T. G. & Steenbergen, W. Review of methodological developments in laser Doppler flowmetry. *Lasers Med Sci* **24**, 269-283 (2009).
3. Newton, D. J., Khan, F. & Belch, J. J. Assessment of microvascular endothelial function in human skin. *Clin Sci (Lond)* **101**, 567-572 (2001).
4. Barcelos, A., Lamas, C. & Tibirica, E. Evaluation of microvascular endothelial function in patients with infective endocarditis using laser speckle contrast imaging and skin video-capillaroscopy: research proposal of a case control prospective study. *BMC Res Notes* **10**, 342 (2017).
5. Wang-Evers, M. et al. Assessing the impact of aging and blood pressure on dermal microvasculature by reactive hyperemia optical coherence tomography angiography. *Sci Rep* **11**, 13411 (2021).
